# Glycemic Index and Glycemic Load Estimates in the Dietary Approach of Polycystic Ovary Syndrome

**DOI:** 10.3390/nu15153483

**Published:** 2023-08-07

**Authors:** Aspasia Manta, Stavroula A. Paschou, Georgia Isari, Ioanna Mavroeidi, Sophia Kalantaridou, Melpomeni Peppa

**Affiliations:** 1 Endocrine Unit, 2nd Propaedeutic Department of Internal Medicine, Research Institute and Diabetes Center, Attikon University Hospital, School of Medicine, National and Kapodistrian University of Athens, 12641 Athens, Greece; aspa.manta@gmail.com (A.M.); georgiaisari@gmail.com (G.I.); joannamavroeidi@gmail.com (I.M.); 2 Endocrine Unit and Diabetes Center, Department of Clinical Therapeutics, Alexandra Hospital, School of Medicine, National and Kapodistrian University of Athens, 11528 Athens, Greece; s.a.paschou@gmail.com; 3 Department of Obstetrics and Gynecology, Attikon University Hospital, School of Medicine, National and Kapodistrian University of Athens, 12641 Athens, Greece; sophiakalantaridou@gmail.com

**Keywords:** polycystic ovary syndrome, anovulation, hyperandrogenism, menstrual disorders, quality of life, nutrition, inflammation, oxidative stress, diet, carbohydrates, insulin resistance, glycemic load, glycemic index, dietary advanced glycation end products

## Abstract

Polycystic ovary syndrome is a common endocrine disorder characterized by hormonal imbalances and various metabolic abnormalities linked to insulin resistance via a vicious cycle. Genetic and environmental factors underlie its pathogenesis and evolution. Nutrition, in terms of nutrient composition, dietary patterns, endocrine-disrupting chemicals, and food processing and preparation, has gained significant attention in the pathogenesis and the therapeutic approach of polycystic ovary syndrome. Carbohydrate intake seems to be a critical point in the diet assignment. Glycemic index and glycemic load constitute indexes of the impacts of dietary carbohydrates on postprandial glucose levels. Numerous studies have indicated that a high glycemic index and glycemic load diet may exacerbate insulin resistance, a key feature of the syndrome, and offer a risk for its development and its complications. Conversely, low-glycemic index and low-glycemic load diets seem to improve insulin sensitivity, regulate menstrual cycles, and mitigate the risk of comorbidities associated with polycystic ovary syndrome, such as obesity, alterations in body composition, type 2 diabetes, cardiovascular disease, and quality of life. This comprehensive review aims to explore the relevance of nutrition and more specifically, the association of glycemic index and glycemic load with the various aspects of polycystic ovary syndrome, as well as to assess the potential benefits of manipulating those indexes in the dietary approach for the syndrome.

## 1. Introduction

Polycystic ovary syndrome (PCOS) is a complex, polygenic metabolic condition and the most common endocrine disorder in women of reproductive age. According to the 2003 Rotterdam criteria, PCOS is defined as clinical or biochemical hyperandrogenism, an indication of oligo-anovulation and polycystic-appearing ovarian morphology on the ultrasound, excluding any other relevant conditions [1,2]. Currently, four recognized phenotypes of PCOS include all possible combinations of these characteristics: (1) hyperandrogenism, oligo-anovulation and polycystic ovarian morphology; (2) hyperandrogenism and oligo-anovulation with normal ovarian morphology; (3) hyperandrogenism and polycystic ovarian morphology with normal ovulation; and (4) polycystic ovarian morphology and oligo-anovulation without the presence of hyperandrogenism [3]. The most common PCOS symptoms are hirsutism, alopecia, and acne, all linked to hyperandrogenism, as well as menstrual irregularities, including oligomenorrhea and amenorrhea [3,4].

The key factor in the development of PCOS, which contributes to increased androgen synthesis and the clinical and biochemical symptoms of the condition, is a malfunction in the ovary, due to both genetic and environmental factors [3,5]. Adiposity, especially abdominal, has been thoroughly studied as a risk factor for the onset and progression of PCOS [6]. In general, low-grade inflammation, oxidative stress (OS), insulin resistance (IR), and hyperandrogenism form a vicious cycle that constitutes the basic underlying pathophysiological mechanism in PCOS [7,8,9]. Women with PCOS have an increased risk of type 2 diabetes, obesity, and long-term cardiovascular (CV) complications. Glucose abnormalities are especially prominent in obese women with PCOS [10], as well as in those with menstrual irregularities [4]. PCOS women are also more likely to experience mild and serious anxiety and depressive symptoms, which negatively affect their quality of life [11,12].

Aside from these well-known predisposing factors, the pathophysiology and evolution of PCOS appear to be heavily influenced by dietary habits, diet composition, food processing and preparation, and endocrine-disrupting chemicals, which may impair ovarian function either directly or indirectly through IR, inflammation, and OS induction [6,13,14].

A specific dietary compound, the dietary advanced glycation end products (dAGE), seems to be involved in health and disease, including PCOS [15,16,17,18,19]. Advanced glycation end products (AGE) are formed constantly in the body by the nonenzymatic glycation of proteins, lipids, and nucleic acids. This process is accelerated in conditions characterized by hyperglycemia, dyslipidemia, OS, and IR [17]. The total body AGE pool is greatly influenced by dAGE consumption, and methods of food processing are major determinants [20].

Another important dietary compound, carbohydrates (CHO), has been linked to chronic inflammation, IR, and CV morbidity in PCOS women [21].

The approaches used in PCOS treatment are mostly determined by the intended outcome. Fertility, control of menstrual irregularities, improvement of hyperandrogenism, enhancement of insulin sensitivity, and weight loss are some of the main targets that are particularly important [1,2,22]. The treatment options include pharmacologic treatment with metformin, oral contraceptives and antiandrogens, estrogen receptor modulators such as clomifene for women who are interested in becoming pregnant, exogenous gonadotropins, or even laparoscopic surgical procedures [1,2,22].

Lifestyle modifications are currently considered one of the main treatment approaches for women with PCOS. The International Evidence-Based Guideline for the Assessment and Management of PCOS has highlighted the significance of nutrition in PCOS and suggested dietary and exercise interventions as the first line of treatment, independently of weight status [22].

The Mediterranean and ketogenic diets appear to be particularly beneficial in terms of weight loss, body composition, and metabolic parameters, including blood glucose levels and IR and a number of cardiometabolic abnormalities and hormonal imbalances, even in slim PCOS patients [23,24,25,26]. Following the publication of a list detailing the AGE contents of numerous staple foods [20], dietary changes in the form of designing low-dAGE diets have been found valuable [17,18,19]. In addition, modification of CHO consumption seems to have a positive impact on several elements of PCOS pathogenesis and may be one of the key strategies for treating these patients [21,22].

The glycemic index (GI) and the glycemic load (GL) are two important dietary indexes, which reflect the CHO effects in the body. The GI estimates the impact of any diet rich in CHO on postprandial blood glucose levels. Greater CHO absorption leads to higher postprandial glucose levels and a high GI. The GL considers both the GI and the quantity of CHOs and seems to be a more accurate indicator of a food’s impact on blood glucose and insulin levels than the GI alone [27]. High-GI and high-GL diets have been associated with a number of chronic conditions [27]. Diets with elevated GI and GL are linked to a greater risk of type 2 diabetes, CV disease, and stroke, which are even more pronounced in overweight and obese patients [28]. Lastly, there is substantial evidence that GI and GL are associated with different types of hormones and non-hormone related cancers [29,30].

In practical terms, existing lists provide data on the serving sizes, CHO content, and GI and GL indexes of a variety of foods. These lists can be used in order to design dietary patterns according to GI and GL values [31].

**Figure 1:**
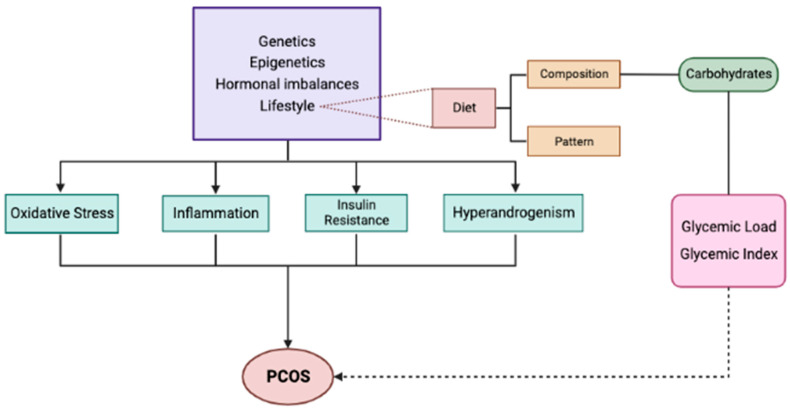
Etiology and underlying mechanisms of PCOS.

The main causes and pathogenetic mechanisms of PCOS are presented in Figure 1.

In the present review, we will try to identify the association of dietary GI and GL indexes with various pathophysiological aspects of PCOS and its evolution.

## 2. Effects of Dietary GI and GL Indexes on PCOS Risk

In addition to genetic and environmental factors, hyperinsulinemia due to IR, diabetes mellitus, obesity, family history for PCOS among first degree relatives, premature adrenarche, fetal androgen exposure, and low birth weight all constitute risk factors for PCOS [1,2,22]. Nutrition is considered another important determinant of PCOS risk, in terms of dietary patterns and specific nutrient intakes [32]. the amount of CHOs has especially been linked to increased PCOS risk [33].

Focusing on the dietary GI and GL indexes, it seems that women who follow high-GI and high-GL diets are more likely to develop PCOS [34,35]. According to a recent observational study, even the consumption of medium GI products increases the probability of PCOS by more than three-fold [36].

In addition, numerous studies have shown that PCOS patients consume noticeably more foods commonly high in GI or GL than healthy controls [35,36,37,38,39]. In comparison to patients of normal weight, obese PCOS patients consume foods with a higher mean dietary GI [40]. Obese and the classic PCOS phenotype are even considered age-independent predictors of higher dietary GI [39].

## 3. Effect of Dietary GI and GL Indexes on PCOS Pathophysiology

### 3.1. Effect of Dietary GI and GL Indexes on Glucose and Insulin Homeostasis

Glucose homeostasis in the whole body is dependent on the insulin secretion and action. IR is a complex phenomenon, due to its molecular and cellular aspects, leading to the disruption of insulin metabolism and elevated glucose levels. IR has been extensively studied and has been linked to numerous cardiometabolic and cognitive disorders and cancer [41,42,43].

IR can be driven by a number of hereditary and lifestyle factors, with diet being one of the main contributors. Macronutrient composition, particularly the intakes of CHOs, proteins, and fats, can influence insulin sensitivity. High-CHO diets, especially those rich in processed CHOs and sugars, may trigger rapid spikes in blood glucose levels, leading to IR. Dietary fiber consumption is also essential for regulating insulin metabolism. Whole grains, fruits, vegetables, and legumes are examples of high-fiber diets that slow down the digestion and absorption of carbs, reducing blood sugar rises and promoting improved insulin regulation [44,45].

IR is one of the major pathophysiologic mechanisms implicated in PCOS and can be present regardless of adiposity, body composition, and androgen levels [1,2,6,46]. Overall, IR is present in 75% of lean women and 95% of obese women with PCOS [46]. IR can exacerbate PCOS symptoms, leading to even more difficulties in weight management, excessive production of androgens, and disrupted ovulation [1,2,5,6].

The effects of different dietary patterns on glucose and insulin homeostasis in PCOS patients have been studied considerably. An overly high-fat diet, especially one high in saturated fat, raises the likelihood of developing IR and worsens its negative consequences [47]. CHOs have been linked to disturbed insulin homeostasis and IR, whereas a modest decrease in dietary CHO content was found to reduce fasting glucose, insulin, and IR while increasing insulin sensitivity [48,49,50]. Reduced dietary CHO consumption also appears to affect pancreatic β-cell responsiveness, which is a measure of insulin secretion, by increasing the first-phase response and decreasing the basal β-cell response [48].

Focusing on GI and GL indexes, there is a clear association between high dietary GI and GL values, insulin levels, and IR, as expressed by the Homeostatic Model Assessment for Insulin Resistance (HOMA-IR) [36]. Women with PCOS and IR were found to consume more GL, compared to those without IR [51].

In regards to dietary interventions, adherence to a low-GI diet was linked to a significant increase in insulin sensitivity [52,53] and a reduction in insulin levels [54], as well as a reduction in HOMA-IR [55], whereas a short-term low-GI intervention had no effect on measures of glycemia [52]. The Dietary Approaches to Stop Hypertension (DASH diet), which is actually a low-GI diet, was initially proposed for the management of hypertension [56]. This diet was studied in overweight and obese women with PCOS and was found to lead to a significant reduction in insulin levels and HOMA-IR [57,58,59], as well as an increase in the quantitative insulin sensitivity check index [59]. Alongside calorie-restricting diets, the DASH diet might be one of the most effective options for reducing IR in PCOS patients [14].

### 3.2. Effects of Dietary GI and GL Indexes on Inflammation Biomarkers

Chronic inflammation refers to a persistent state of subclinical low-grade inflammation in the body that can promote the development and progression of various diseases, including CV disease, cognitive dysfunction, and certain malignancies [60,61].

Diet plays a crucial role in either promoting or mitigating chronic inflammation. Poor nutritional habits have been recognized as a component of the environmental triggers for chronic inflammation, due to their link to the abnormal activation of the innate immune system, resulting in low-grade systemic inflammation [62,63].

Food intake is known to cause a postprandial inflammatory reaction, the extent of which correlates with the level of IR. The caloric and CHO contents, as well as the lipid profile of a meal, are some of the nutrient-dependent parameters that affect postprandial inflammation [64]. Particularly complex CHOs are associated with inflammation through abnormal postprandial rises in glucose and lipids [32]. Dietary intakes of fat, protein, cholesterol, and sodium have also been found to be positively correlated with high-sensitive C-reactive protein (hs-CRP) levels, whereas a low-fiber diet is associated with increased inflammation [65].

Obesity is also a key factor for low-grade chronic inflammation, independently of specific nutrients, through a mechanism that involves adipocytes, macrophages, and the expression of pro-inflammatory receptors [66].

Chronic low-grade inflammation is considered to be involved in PCOS pathogenesis. A number of studies have found increased CRP levels in PCOS patients, although it is still unclear whether the inflammation is brought on by PCOS itself or by IR and obesity [67]. Inflammation is a cause of hyperandrogenism and can also exacerbate IR and affect ovulation via several pathways involving pro-inflammatory compounds [5].

Focusing on the dietary GI and GL indexes, the combination of high-protein and low-GL foods caused a significant decrease in hs-CRP levels, compared to a standard hypocaloric diet [54]. In overweight and obese women with PCOS, the DASH diet, a classical low-GI diet, resulted in a significant reduction in hs-CRP levels [57]. Adherence to a low-GI diet for 3 months was found to substantially decrease inflammation, as indicated by rising uric acid levels and glutathione peroxidase activity [68].

### 3.3. Effects of Dietary GI and GL Indexes on Oxidative Stress Biomarkers

An imbalance between pro-oxidants and antioxidants leads to oxidative stress (OS). Oxidative compounds, such as reactive oxygen species (ROS) and reactive nitrogen species (RNS), are involved in a number of processes, including those that regulate signaling, cell growth, and differentiation. Excess ROS accumulation can induce cell, protein, and lipid damage [5,65].

In a similar way to low-grade systemic inflammation, OS can be induced by postprandial hyperglycemia and is highly influenced by eating habits. Among the pro-inflammatory dietary patterns, CHOs are deemed responsible for the induction of OS, as demonstrated by large studies focusing on different populations [32]. On the other hand, dietary modifications can have an impact on the redox state, particularly in individuals with diabetes, hypertension, obesity, or dyslipidemia. Dietary antioxidants, hypocaloric diets that cause adipose tissue loss, and the substitution of animal protein for plant protein all enhance the antioxidant status of patients with these disorders [69].

Various circulating biomarkers of OS are abnormal in PCOS patients, regardless of weight status, indicating that OS plays a role in the etiology of the condition [70]. PCOS patients have markedly greater levels of homocysteine, malondialdehyde, asymmetric dimethylarginine, and superoxide dismutase activity, compared to healthy women. They also have lower levels of glutathione and paraoxonase-1 activity [70]. However, the mechanism of this link is not entirely clear, considering that PCOS patients frequently present with IR, obesity, and hyperandrogenemia, which also promote OS [71]. Nutritional supplementation is considered beneficial for OS related to PCOS and its accompanying disorders. Vitamin D, flavonoids, selenium, probiotics, vitamin E, folate, and omega-3 fatty acids are among the supplements being studied for their capacity to decrease OS and its detrimental effects on patients’ hormonal and lipid profiles [72].

Regarding the GI and GL indexes, the low GI DASH diet seems to have antioxidant properties that increase the total antioxidant capacity, 2,2-Diphenyl-1-picrylhydrazyl (DPPH) activity, glutathione, and nitric oxide [58,59,73] and decrease malondialdehyde levels [59].

### 3.4. Effects of Dietary GI and GL Indexes on Androgen Levels

Sex hormones are essential for growth, sexual development, and reproduction. They are also associated with metabolic parameters and relevant diseases. Low levels of circulating androgens have been associated with obesity and visceral adiposity in males, whilst excessive levels of androgens have also been connected to metabolic abnormalities in women. According to research, nutrition is known to influence androgen levels. A particular nutrient’s effects or other metabolic pathways, such as diet-induced changes in the context of obesity or IR, may mediate such an effect [74]. Regarding nutritional patterns, intermittent fasting was found to be beneficial for lowering androgen levels (testosterone and the free androgen index (FAI)) while raising sex hormone-binding globulin (SHBG) levels in premenopausal obese women [75].

The majority of PCOS patients with oligo-amenorrhea also have biochemical hyperandrogenemia, with the ovaries being the primary source of this androgen excess [3].

Androgen production is directly influenced by pro-inflammatory stimuli, while biomarkers of OS and inflammation are closely linked to circulating androgens [1,2,3,6]. In PCOS women, insulin levels have an impact on circulating androgens, independently of changes in gonadotropin secretion [76]. Nevertheless, ovarian androgen production in PCOS patients arising from dietary-induced inflammation may not be reliant on excessive body fat or IR [6]. Various dietary interventions have been promising in regulating androgen levels in PCOS patients. In obese PCOS individuals with anovulatory infertility, a hypocaloric high-protein diet and an exercise regimen decreased serum androgens, namely SHBG, androstenedione, and dehydroepiandrosterone sulphate [77].

However, the association of dietary GI and GL indexes specifically with androgen levels in PCOS is not clear. In overweight women with PCOS, a low-GI diet resulted in a significant reduction in total testosterone and an increase in sex hormone-binding globulin [38], while a reduction in dietary CHO content also significantly reduced total testosterone [48]. The low-GI DASH diet had a significant reduction in serum androstenedione [73] and significant increases in sex hormone-binding globulin [59,73] and anti-Müllerian hormone (AMH) [59]. A low-GI diet had a significant but similar decrease in testosterone in obese PCOS patients, compared to the effects of a conventional hypocaloric diet [54].

On the contrary, a 3-month low-GI diet intervention had no effect on androgen levels [78]. Wong et al. compared the impact of a low-GL diet with a low-fat diet in overweight and obese adolescents with PCOS and also found no difference in testosterone after either intervention [79].

### 3.5. Effects of Dietary GI and GL Indexes on Weight Status

Obesity is a serious public health issue that affects both children and adults, with a high prevalence globally.

This trend is also seen in PCOS, as 30% to 70% of women are overweight or obese. Abdominal obesity, in particular, affects and accentuates all metabolic and reproductive manifestations of PCOS [80].

Weight loss is recommended as an essential component of treatment for PCOS patients with an elevated BMI. Physical activity and maintenance of a healthy weight status are crucial components of the treatment of metabolic dysfunction related to PCOS [2,3,6]. Adherence to a hypocaloric diet and weight reduction of as little as 5% have been shown to improve clinical, metabolic, and reproductive abnormalities [81,82]. In women with PCOS, improved fatty acid oxidation, weight loss, and prevention of further weight gain may result from dietary changes that lower postprandial hyperglycemia and hyperinsulinemia [83].

Independent of calorie restriction, a reduction in CHO consumption has been associated with a greater decrease in adipose tissue and an impact on body composition [49]. These impacts may be related to changes in insulin release [84]. A reduced CHO diet for 8 weeks in women with PCOS significantly increased body fat loss, compared to the standard diet. The diet induced a decrease in subcutaneous-abdominal, intra-abdominal, and thigh-intermuscular adipose tissues [49]. Previous research also showed that females with PCOS had a lower risk of being overweight and obese when their daily consumption of plant protein was increased by 10 gr [85].

Regarding the GI and GL indexes, healthy but also PCOS women consuming high-GI or high-GL diets were found to have a higher BMI and waist circumference [38,39]. A significant inverse association between dietary GL and waist-to-hip ratio in women with PCOS was also reported [86].

In PCOS women, a low-GI diet decreased body mass, BMI, and waist, hip, and arm circumferences [87,88] and affected several indexes of body compositions [87]. A vegan low-GI diet had a greater decrease in energy and fat intake and a significant weight loss at 3 months, but not at 6 months, compared to conventional low-calorie diets [89]. The low-GI DASH diet appears to be an effective dietary strategy, as it was found to considerably reduce body weight, BMI [58,59], waist and hip circumferences [57], and fat mass [73], even when compared to a standard calorie-restricting diet [58].

Nevertheless, several studies reported an increased adherence to a low-GI diet quite similar to other diets, whether hypocaloric [54], low-fat [79], or designed to decrease hypercholesterolemia [55].

## 4. Effects of Dietary GI and GL Indexes on PCOS Phenotype

Menstrual cycles are significantly influenced not only by hormonal factors but also adiposity and body composition. Irregular menstrual cycles substantially correlate with both total and central obesity. Hormonal variables, notably insulin and SHBG, seem to have significant impacts on this connection [1,2,3,4,6,10].

Menstrual irregularity is one of the main features of PCOS and an indication of dysfunctional ovulation [1,2,3,4,22]. Nutrition is a key factor that affects reproduction directly through hyperglycemia or indirectly through altered insulin sensitivity and androgen levels, not only in terms of caloric excess but also in terms of the type of dietary intake. Specific nutrients, including CHOs or trans fatty acids, affect ovarian function and may influence the menstrual cycle [90]. A higher CHO intake has been linked to an increased risk of infertility caused by anovulation [91].

On the other hand, weight loss improved menstrual function and ovulatory patterns in the majority of women, and in obese PCOS individuals’, normal menstrual function and fertility were restored with the improvement of insulin sensitivity [92]. Regarding eating habits, obese PCOS individuals with anovulatory infertility significantly increased fertility with a hypocaloric low-protein diet and an exercise program [77]. In addition to dietary and lifestyle changes, using a probiotic supplement effectively regularized the menstrual cycle in PCOS patients [77].

A low-GI diet was found to improve menstrual regularity in overweight and obese women with PCOS [38,53] and increased the ovulatory cycles, compared to a normal-GI diet [93]. In overweight women with PCOS, a low-GI diet also resulted in a significant decrease in acne occurrence [38].

## 5. Effects of Dietary GI and GL Indexes on CV System

Overall, nutrition plays a major role in CV health and associated disorders. Regarding specific nutrients, high-CHO diets are associated with increased CV and all-cause mortality after 10 years [94]. However, higher intakes of CHOs from fruit and legumes are linked to decreased rates of CV and all-cause death, suggesting that the quality of CHOs may affect this connection [95].

Elevated levels of plasma triglycerides and decreased high-density lipoprotein cholesterol constitute the characteristic lipid profile in PCOS that is more profound in obese patients, while low-density lipoprotein is usually only slightly increased [96].

Concerning dietary patterns, research has found that the consumption of high-CHO diets could increase triglycerides dose-dependently [97], whereas low-CHO diets could be effective in decreasing triglycerides [98]. In PCOS patients, a reduction in dietary CHO content was found to significantly reduce all cholesterol measures but not triglycerides [48]. In terms of lipids, a diet reduced in fat, particularly saturated fatty acids (SFA), had a good effect on the metabolic profile [57], whereas cholesterol demonstrated a positive association with CRP levels in lean women with PCOS [99]. Additionally, researchers discovered that increasing the intake of polyunsaturated fatty acids (PUFA) in PCOS patients who undertook a three-month dietary intervention was beneficial for glucose levels [100].

However, existing research findings on the effects of a low-GI or low-GL diets on lipid levels are contradictory. Short-term low-GI diet adherence resulted in a reduction in non-esterified fatty acid levels [52], with no substantial effect on the total lipid profile [52,53]. Longer interventions seem to be more effective. Following a low-GI diet for 3 and 4 months resulted in considerable reductions in fatty acids [78] and low-density cholesterol [54], respectively. In the latter study, the low-GI diet was found to be superior to a typical hypocaloric diet [54]. The DASH diet was compared to a conventional calorie-restricting diet for 8 weeks and was found to significantly decrease serum triglyceride and very-low-density lipoprotein cholesterol levels [58].

## 6. Effects of Dietary GI and GL Indexes on Symptoms and Quality of Life

The association between diet and quality of life is widely recognized and extensively studied. A balanced and nutritious diet plays a vital role in promoting overall well-being and improving one’s quality of life.

PCOS has been reported to have a generally negative effect on health-related quality of life (HRQOL). PCOS patients experience elevated signs of anxiety and depression linked to their condition [101]. Obesity and hyperandrogenemia enhance the likelihood of depression and food cravings, creating a vicious cycle where obesity and metabolic syndrome get progressively worse [102].

Both treatment for PCOS and weight loss interventions were found to improve in HRQOL parameters, and their combination seemed to be the most effective managing strategy [101]. Probiotic supplements and dietary and lifestyle changes also had a substantial impact on PCOS women’s quality of life [103].

Regarding the issue of dietary plans based on GI and GL indexes, a low-GI diet was associated with less hunger [104] and a significant reduction in hypoglycemia symptoms [88]. A low-GI diet also increased quality of life scores in PCOS patients; however, the results were comparable with the Therapeutic Lifestyle Changes diet, a standard healthy diet designed for the management of hypercholesterolemia [55]. Feelings of fullness were greater in the early postprandial phase after a high-GL meal, but no differences were observed in the late postprandial phase [104].

**Figure 2:**
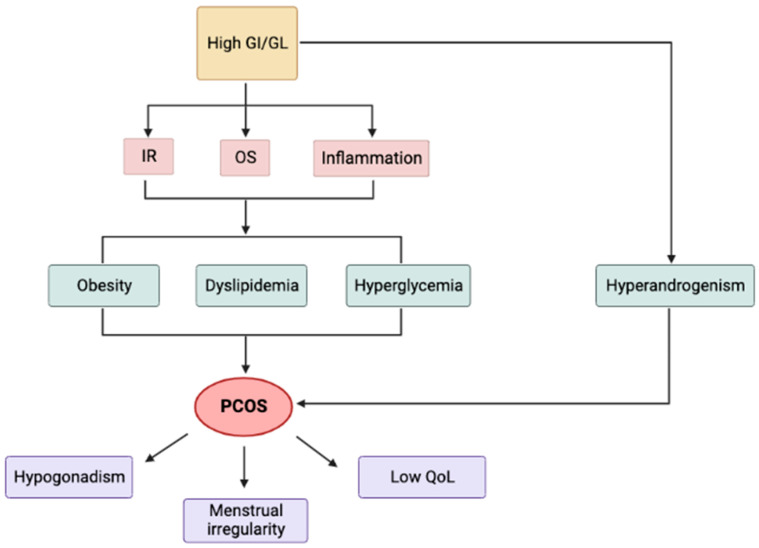
Effects of GI and GL on PCOS. GI: glycemic index; GL: glycemic load; IR: insulin resistance; OS: oxidative stress; QoL: quality of life.

A summary of the effects of GI and GL on PCOS Is presented in Figure 2.

## 7. Conclusions

The nutritional approach for PCOS patients has drawn significant attention, and there is growing interest in the roles of GI and GL as potential tools in its management. PCOS is characterized by IR, hormonal imbalances and metabolic changes, poor quality of life, and cardiovascular disease. The existing data suggest that GI and GL indexes might be useful in the dietary approach of patients with risk factors or a certain diagnosis of PCOS. High-GI and high-GL diets seem to induce systemic inflammation, OS, and IR and might interfere with PCOS symptoms, poor quality of life, and increased cardiovascular risk, all of them being more pronounced in obese patients. On the other hand, consumptions of low-GI and low-GL diets seem to alleviate IR, regulate menstrual cycles, and mitigate the risk of comorbidities associated with PCOS. Continued research is crucial to refine dietary recommendations regarding the cost-effectiveness of estimating the GI and GL indexes in the dietary approach of PCOS patients, especially regarding the effectiveness in the long-term consequences, such as cardiovascular, psychological, and quality of life.

## 8. Future Directions

Further research in the context of the role of nutrition in PCOS is essential in order to expand our understanding about the precise dietary interventions that can effectively change the natural history of the disease and the rate of complications. The ideal macronutrient composition, the need for specific micronutrients, and the effects of particular dietary patterns on PCOS symptoms, such as CHOs, are all areas of ongoing research.

Despite the several positive effects of low-GI and low-GL diets on different metabolic and other parameters, as described above, additional research on these indexes in PCOS is also essential. Future studies could investigate the long-term effects of low-GI/GL diets on hormonal balance, weight management, and metabolic parameters in women with PCOS. Beyond that, exploring the individual variability in glycemic response among PCOS patients and understanding the influence of factors such as genetics, gut microbiota, and lifestyle choices would provide valuable insights. A solid understanding of their effectiveness would also be aided by comparison studies comparing the effects of various dietary methods, such as low-GI/GL diets versus other dietary approaches. Lastly, the long-term sustainability of dietary changes, the potential interactions between diet and medication, and the influence of diet on reproductive outcomes in women with PCOS could be examined.

By conducting further research in this area, we can refine dietary recommendations and develop tailored strategies based on the potential benefits of GI and GL in the management of PCOS, ultimately improving the health and quality of life for affected individuals.

## Data Availability

Data supporting this article are included within the reference list.

## References

[1] Rotterdam ESHRE/ASRM-Sponsored PCOS Consensus Workshop Group (2004). Revised 2003 Consensus on Diagnostic Criteria and Long-Term Health Risks Related to Polycystic Ovary Syndrome (PCOS). Hum. Reprod..

[2] Christ J.P., Cedars M.I. (2023). Current Guidelines for Diagnosing PCOS. Diagnostics.

[3] Azziz R. (2018). Polycystic Ovary Syndrome. Obstet. Gynecol..

[4] Christodoulopoulou V., Trakakis E., Pergialiotis V., Peppa M., Chrelias C., Kassanos D., Papantoniou N. (2016). Clinical and Biochemical Characteristics in PCOS Women With Menstrual Abnormalities. J. Fam. Reprod. Health.

[5] Sadeghi H.M., Adeli I., Calina D., Docea A.O., Mousavi T., Daniali M., Nikfar S., Tsatsakis A., Abdollahi M. (2022). Polycystic Ovary Syndrome: A Comprehensive Review of Pathogenesis, Management, and Drug Repurposing. Int. J. Mol. Sci..

[6] González F. (2015). Nutrient-Induced Inflammation in Polycystic Ovary Syndrome: Role in the Development of Metabolic Aberration and Ovarian Dysfunction. Semin. Reprod. Med..

[7] Shorakae S., Teede H., de Courten B., Lambert G., Boyle J., Moran L. (2015). The Emerging Role of Chronic Low-Grade Inflammation in the Pathophysiology of Polycystic Ovary Syndrome. Semin. Reprod. Med..

[8] Genazzani A.D., Genazzani A.R. (2023). Polycystic Ovary Syndrome as Metabolic Disease: New Insights on Insulin Resistance. Eur. Endocrinol..

[9] Mohammadi M. (2019). Oxidative Stress and Polycystic Ovary Syndrome: A Brief Review. Int. J. Prev. Med..

[10] Trakakis E., Basios G., Peppa M., Simeonidis G., Labos G., Creatsa M., Misailidou M., Boutati E., Vaggopoulos V., Panagopoulos P. (2012). The Prevalence of Glucose Metabolism Abnormalities in Greek Women with Polycystic Ovary Syndrome. Gynecol. Endocrinol..

[11] Stefanaki C., Bacopoulou F., Livadas S., Kandaraki A., Karachalios A., Chrousos G.P., Diamanti-Kandarakis E. (2015). Impact of a Mindfulness Stress Management Program on Stress, Anxiety, Depression and Quality of Life in Women with Polycystic Ovary Syndrome: A Randomized Controlled Trial. Stress.

[12] Livadas S., Chaskou S., Kandaraki A.A., Skourletos G., Economou F., Christou M., Boutzios G., Karachalios A., Zerva A., Xyrafis X. (2011). Anxiety Is Associated with Hormonal and Metabolic Profile in Women with Polycystic Ovarian Syndrome. Clin. Endocrinol..

[13] Diamanti-Kandarakis E., Papalou O., Kandaraki E.A., Kassi G. (2017). MECHANISMS IN ENDOCRINOLOGY: Nutrition as a Mediator of Oxidative Stress in Metabolic and Reproductive Disorders in Women. Eur. J. Endocrinol..

[14] Shang Y., Zhou H., Hu M., Feng H. (2020). Effect of Diet on Insulin Resistance in Polycystic Ovary Syndrome. J. Clin. Endocrinol. Metab..

[15] Cai W., Gao Q.-D., Zhu L., Peppa M., He C., Vlassara H. (2002). Oxidative Stress-Inducing Carbonyl Compounds from Common Foods: Novel Mediators of Cellular Dysfunction. Mol. Med..

[16] Uribarri J., Cai W., Sandu O., Peppa M., Goldberg T., Vlassara H. (2005). Diet-Derived Advanced Glycation End Products Are Major Contributors to the Body’s AGE Pool and Induce Inflammation in Healthy Subjects. Ann. N. Y. Acad. Sci..

[17] Peppa M., Uribarri J. (2017). Dietary AGEs and Their Role in Health and Disease.

[18] Peppa M., Mavroeidi I. (2021). Experimental Animal Studies Support the Role of Dietary Advanced Glycation End Products in Health and Disease. Nutrients.

[19] Rutkowska A., Diamanti-Kandarakis E. (2016). Do Advanced Glycation End Products (AGEs) Contribute to the Comorbidities of Polycystic Ovary Syndrome (PCOS)?. Curr. Pharm. Des..

[20] Goldberg T., Cai W., Peppa M., Dardaine V., Baliga B.S., Uribarri J., Vlassara H. (2004). Advanced Glycoxidation End Products in Commonly Consumed Foods. J. Am. Diet. Assoc..

[21] Zhang X., Zheng Y., Guo Y., Lai Z. (2019). The Effect of Low Carbohydrate Diet on Polycystic Ovary Syndrome: A Meta-Analysis of Randomized Controlled Trials. Int. J. Endocrinol..

[22] Teede H.J., Misso M.L., Costello M.F., Dokras A., Laven J., Moran L., Piltonen T., Norman R.J., Andersen M., Azziz R. (2018). Recommendations from the International Evidence-Based Guideline for the Assessment and Management of Polycystic Ovary Syndrome. Fertil. Steril..

[23] Cincione I.R., Graziadio C., Marino F., Vetrani C., Losavio F., Savastano S., Colao A., Laudisio D. (2022). Short-Time Effects of Ketogenic Diet or Modestly Hypocaloric Mediterranean Diet on Overweight and Obese Women with Polycystic Ovary Syndrome. J. Endocrinol. Investig..

[24] Paoli A., Mancin L., Giacona M.C., Bianco A., Caprio M. (2020). Effects of a Ketogenic Diet in Overweight Women with Polycystic Ovary Syndrome. J. Transl. Med..

[25] Barrea L., Arnone A., Annunziata G., Muscogiuri G., Laudisio D., Salzano C., Pugliese G., Colao A., Savastano S. (2019). Adherence to the Mediterranean Diet, Dietary Patterns and Body Composition in Women with Polycystic Ovary Syndrome (PCOS). Nutrients.

[26] Barrea L., Frias-Toral E., Verde L., Ceriani F., Cucalón G., Garcia-Velasquez E., Moretti D., Savastano S., Colao A., Muscogiuri G. (2021). PCOS and Nutritional Approaches: Differences between Lean and Obese Phenotype. Metabol. Open.

[27] Jayedi A., Soltani S., Jenkins D., Sievenpiper J., Shab-Bidar S. (2022). Dietary Glycemic Index, Glycemic Load, and Chronic Disease: An Umbrella Review of Meta-Analyses of Prospective Cohort Studies. Crit. Rev. Food Sci. Nutr..

[28] Hardy D.S., Garvin J.T., Xu H. (2020). Carbohydrate Quality, Glycemic Index, Glycemic Load and Cardiometabolic Risks in the US, Europe and Asia: A Dose–Response Meta-Analysis. Nutr. Metab. Cardiovasc. Dis..

[29] Sieri S., Krogh V. (2017). Dietary Glycemic Index, Glycemic Load and Cancer: An Overview of the Literature. Nutr. Metab. Cardiovasc. Dis..

[30] George S.M., Mayne S.T., Leitzmann M.F., Park Y., Schatzkin A., Flood A., Hollenbeck A., Subar A.F. (2008). Dietary Glycemic Index, Glycemic Load, and Risk of Cancer: A Prospective Cohort Study. Am. J. Epidemiol..

[31] Carneiro L., Leloup C. (2020). Mens Sana in Corpore Sano: Does the Glycemic Index Have a Role to Play?. Nutrients.

[32] Barrea L., Marzullo P., Muscogiuri G., Di Somma C., Scacchi M., Orio F., Aimaretti G., Colao A., Savastano S. (2018). Source and Amount of Carbohydrate in the Diet and Inflammation in Women with Polycystic Ovary Syndrome. Nutr. Res. Rev..

[33] Eslamian G., Hekmatdoost A. (2019). Nutrient Patterns and Risk of Polycystic Ovary Syndrome. J. Reprod. Infertil..

[34] Panjeshahin A., Salehi-Abargouei A., Anari A.G., Mohammadi M., Hosseinzadeh M. (2020). Association between Empirically Derived Dietary Patterns and Polycystic Ovary Syndrome: A Case-Control Study. Nutrition.

[35] Eslamian G., Baghestani A.-R., Eghtesad S., Hekmatdoost A. (2017). Dietary Carbohydrate Composition Is Associated with Polycystic Ovary Syndrome: A Case-Control Study. J. Hum. Nutr. Diet..

[36] Mizgier M., Jarząbek-Bielecka G., Formanowicz D., Jodłowska-Siewert E., Mruczyk K., Cisek-Woźniak A., Kędzia W., Opydo-Szymaczek J. (2021). Dietary and Physical Activity Habits in Adolescent Girls with Polycystic Ovary Syndrome (PCOS)-HAstudy. J. Clin. Med..

[37] Altieri P., Cavazza C., Pasqui F., Morselli A.M., Gambineri A., Pasquali R. (2013). Dietary Habits and Their Relationship with Hormones and Metabolism in Overweight and Obese Women with Polycystic Ovary Syndrome. Clin. Endocrinol..

[38] Shishehgar F., Mirmiran P., Rahmati M., Tohidi M., Ramezani Tehrani F. (2019). Does a Restricted Energy Low Glycemic Index Diet Have a Different Effect on Overweight Women with or without Polycystic Ovary Syndrome?. BMC Endocr. Disord..

[39] Graff S.K., Mário F.M., Alves B.C., Spritzer P.M. (2013). Dietary Glycemic Index Is Associated with Less Favorable Anthropometric and Metabolic Profiles in Polycystic Ovary Syndrome Women with Different Phenotypes. Fertil. Steril..

[40] Barr S., Hart K., Reeves S., Sharp K., Jeanes Y.M. (2011). Habitual Dietary Intake, Eating Pattern and Physical Activity of Women with Polycystic Ovary Syndrome. Eur. J. Clin. Nutr..

[41] Hölscher C. (2020). Brain Insulin Resistance: Role in Neurodegenerative Disease and Potential for Targeting. Expert Opin. Investig. Drugs.

[42] Kosmas C.E., Bousvarou M.D., Kostara C.E., Papakonstantinou E.J., Salamou E., Guzman E. (2023). Insulin Resistance and Cardiovascular Disease. J. Int. Med. Res..

[43] Màrmol J.M., Carlsson M., Raun S.H., Grand M.K., Sørensen J., Lang Lehrskov L., Richter E.A., Norgaard O., Sylow L. (2023). Insulin Resistance in Patients with Cancer: A Systematic Review and Meta-Analysis. Acta Oncol..

[44] Mirabelli M., Russo D., Brunetti A. (2020). The Role of Diet on Insulin Sensitivity. Nutrients.

[45] Martins F.O., Conde S.V. (2022). Impact of Diet Composition on Insulin Resistance. Nutrients.

[46] Stepto N.K., Cassar S., Joham A.E., Hutchison S.K., Harrison C.L., Goldstein R.F., Teede H.J. (2013). Women with Polycystic Ovary Syndrome Have Intrinsic Insulin Resistance on Euglycaemic-Hyperinsulaemic Clamp. Hum. Reprod..

[47] Faghfoori Z., Fazelian S., Shadnoush M., Goodarzi R. (2017). Nutritional Management in Women with Polycystic Ovary Syndrome: A Review Study. Diabetes Metab. Syndr. Clin. Res. Rev..

[48] Gower B.A., Chandler-Laney P.C., Ovalle F., Goree L.L., Azziz R., Desmond R.A., Granger W.M., Goss A.M., Bates G.W. (2013). Favourable Metabolic Effects of a Eucaloric Lower-Carbohydrate Diet in Women with PCOS. Clin. Endocrinol..

[49] Goss A.M., Chandler-Laney P.C., Ovalle F., Goree L.L., Azziz R., Desmond R.A., Wright Bates G., Gower B.A. (2014). Effects of a Eucaloric Reduced-Carbohydrate Diet on Body Composition and Fat Distribution in Women with PCOS. Metabolism.

[50] Douglas C.C., Gower B.A., Darnell B.E., Ovalle F., Oster R.A., Azziz R. (2006). Role of Diet in the Treatment of Polycystic Ovary Syndrome. Fertil. Steril..

[51] Cutler D.A., Pride S.M., Cheung A.P. (2019). Low Intakes of Dietary Fiber and Magnesium Are Associated with Insulin Resistance and Hyperandrogenism in Polycystic Ovary Syndrome: A Cohort Study. Food Sci. Nutr..

[52] Barr S., Reeves S., Sharp K., Jeanes Y.M. (2013). An Isocaloric Low Glycemic Index Diet Improves Insulin Sensitivity in Women with Polycystic Ovary Syndrome. J. Acad. Nutr. Diet.

[53] Marsh K.A., Steinbeck K.S., Atkinson F.S., Petocz P., Brand-Miller J.C. (2010). Effect of a Low Glycemic Index Compared with a Conventional Healthy Diet on Polycystic Ovary Syndrome. Am. J. Clin. Nutr..

[54] Mehrabani H.H., Salehpour S., Amiri Z., Farahani S.J., Meyer B.J., Tahbaz F. (2012). Beneficial Effects of a High-Protein, Low-Glycemic-Load Hypocaloric Diet in Overweight and Obese Women with Polycystic Ovary Syndrome: A Randomized Controlled Intervention Study. J. Am. Coll. Nutr..

[55] Kazemi M., McBreairty L.E., Zello G.A., Pierson R.A., Gordon J.J., Serrao S.B., Chilibeck P.D., Chizen D.R. (2020). A Pulse-Based Diet and the Therapeutic Lifestyle Changes Diet in Combination with Health Counseling and Exercise Improve Health-Related Quality of Life in Women with Polycystic Ovary Syndrome: Secondary Analysis of a Randomized Controlled Trial. J. Psychosom. Obstet. Gynecol..

[56] Vollmer W.M., Sacks F.M., Ard J., Appel L.J., Bray G.A., Simons-Morton D.G., Conlin P.R., Svetkey L.P., Erlinger T.P., Moore T.J. (2001). Effects of Diet and Sodium Intake on Blood Pressure: Subgroup Analysis of the DASH-Sodium Trial. Ann. Intern. Med..

[57] Asemi Z., Esmaillzadeh A. (2015). DASH Diet, Insulin Resistance, and Serum Hs-CRP in Polycystic Ovary Syndrome: A Randomized Controlled Clinical Trial. Horm. Metab. Res..

[58] Asemi Z., Samimi M., Tabassi Z., Shakeri H., Sabihi S.-S., Esmaillzadeh A. (2014). Effects of DASH Diet on Lipid Profiles and Biomarkers of Oxidative Stress in Overweight and Obese Women with Polycystic Ovary Syndrome: A Randomized Clinical Trial. Nutrition.

[59] Foroozanfard F., Rafiei H., Samimi M., Gilasi H.R., Gorjizadeh R., Heidar Z., Asemi Z. (2017). The Effects of Dietary Approaches to Stop Hypertension Diet on Weight Loss, Anti-Müllerian Hormone and Metabolic Profiles in Women with Polycystic Ovary Syndrome: A Randomized Clinical Trial. Clin. Endocrinol..

[60] van’t Klooster C.C., Ridker P.M., Hjortnaes J., van der Graaf Y., Asselbergs F.W., Westerink J., Aerts J.G.J.V., Visseren F.L.J. (2019). The Relation between Systemic Inflammation and Incident Cancer in Patients with Stable Cardiovascular Disease: A Cohort Study. Eur. Heart J..

[61] McGrattan A.M., McGuinness B., McKinley M.C., Kee F., Passmore P., Woodside J.V., McEvoy C.T. (2019). Diet and Inflammation in Cognitive Ageing and Alzheimer’s Disease. Curr. Nutr. Rep..

[62] Galland L. (2010). Diet and Inflammation. Nutr. Clin. Pract..

[63] Grosso G., Laudisio D., Frias-Toral E., Barrea L., Muscogiuri G., Savastano S., Colao A. (2022). Anti-Inflammatory Nutrients and Obesity-Associated Metabolic-Inflammation: State of the Art and Future Direction. Nutrients.

[64] Margioris A.N. (2009). Fatty Acids and Postprandial Inflammation. Curr. Opin. Clin. Nutr. Metab. Care.

[65] Khayyatzadeh S.S., Kazemi-Bajestani S.M.R., Bagherniya M., Mehramiz M., Tayefi M., Ebrahimi M., Ferns G.A., Safarian M., Ghayour-Mobarhan M. (2017). Serum High C Reactive Protein Concentrations Are Related to the Intake of Dietary Macronutrients and Fiber: Findings from a Large Representative Persian Population Sample. Clin. Biochem..

[66] Han J.M., Levings M.K. (2013). Immune Regulation in Obesity-Associated Adipose Inflammation. J. Immunol..

[67] Rudnicka E., Suchta K., Grymowicz M., Calik-Ksepka A., Smolarczyk K., Duszewska A.M., Smolarczyk R., Meczekalski B. (2021). Chronic Low Grade Inflammation in Pathogenesis of PCOS. Int. J. Mol. Sci..

[68] Szczuko M., Zapalowska-Chwyć M., Drozd R. (2019). A Low Glycemic Index Decreases Inflammation by Increasing the Concentration of Uric Acid and the Activity of Glutathione Peroxidase (GPx3) in Patients with Polycystic Ovary Syndrome (PCOS). Molecules.

[69] Ávila-Escalante M.L., Coop-Gamas F., Cervantes-Rodríguez M., Méndez-Iturbide D., Aranda-González I.I. (2020). The Effect of Diet on Oxidative Stress and Metabolic Diseases—Clinically Controlled Trials. J. Food Biochem..

[70] Murri M., Luque-Ramírez M., Insenser M., Ojeda-Ojeda M., Escobar-Morreale H.F. (2013). Circulating Markers of Oxidative Stress and Polycystic Ovary Syndrome (PCOS): A Systematic Review and Meta-Analysis. Hum. Reprod. Update.

[71] Zuo T., Zhu M., Xu W. (2016). Roles of Oxidative Stress in Polycystic Ovary Syndrome and Cancers. Oxidative Med. Cell. Longev..

[72] Dubey P., Reddy S., Boyd S., Bracamontes C., Sanchez S., Chattopadhyay M., Dwivedi A. (2021). Effect of Nutritional Supplementation on Oxidative Stress and Hormonal and Lipid Profiles in PCOS-Affected Females. Nutrients.

[73] Azadi-Yazdi M., Karimi-Zarchi M., Salehi-Abargouei A., Fallahzadeh H., Nadjarzadeh A. (2017). Effects of Dietary Approach to Stop Hypertension Diet on Androgens, Antioxidant Status and Body Composition in Overweight and Obese Women with Polycystic Ovary Syndrome: A Randomised Controlled Trial. J. Hum. Nutr. Diet..

[74] Morisset A.-S., Blouin K., Tchernof A. (2008). Impact of Diet and Adiposity on Circulating Levels of Sex Hormone-Binding Globulin and Androgens. Nutr. Rev..

[75] Cienfuegos S., Corapi S., Gabel K., Ezpeleta M., Kalam F., Lin S., Pavlou V., Varady K.A. (2022). Effect of Intermittent Fasting on Reproductive Hormone Levels in Females and Males: A Review of Human Trials. Nutrients.

[76] Dunaif A., Graf M. (1989). Insulin Administration Alters Gonadal Steroid Metabolism Independent of Changes in Gonadotropin Secretion in Insulin-Resistant Women with the Polycystic Ovary Syndrome. J. Clin. Investig..

[77] Palomba S., Giallauria F., Falbo A., Russo T., Oppedisano R., Tolino A., Colao A., Vigorito C., Zullo F., Orio F. (2008). Structured Exercise Training Programme versus Hypocaloric Hyperproteic Diet in Obese Polycystic Ovary Syndrome Patients with Anovulatory Infertility: A 24-Week Pilot Study. Hum. Reprod..

[78] Szczuko M., Drozd A., Maciejewska D., Zapałowska-Chwyć M., Stachowska E. (2019). Decrease in the Level of Nervonic Acid and Increased Gamma Linolenic Acid in the Plasma of Women with Polycystic Ovary Syndrome after a Three-Month Low-Glycaemic Index and Caloric Reduction Diet. Open Life Sci..

[79] Wong J.M.W., Gallagher M., Gooding H., Feldman H.A., Gordon C.M., Ludwig D.S., Ebbeling C.B. (2016). A Randomized Pilot Study of Dietary Treatments for Polycystic Ovary Syndrome in Adolescents. Pediatr. Obes..

[80] Glueck C.J., Goldenberg N. (2019). Characteristics of Obesity in Polycystic Ovary Syndrome: Etiology, Treatment, and Genetics. Metabolism.

[81] Moran L.J., Noakes M., Clifton P.M., Tomlinson L., Norman R.J. (2003). Dietary Composition in Restoring Reproductive and Metabolic Physiology in Overweight Women with Polycystic Ovary Syndrome. J. Clin. Endocrinol. Metab..

[82] Stamets K., Taylor D.S., Kunselman A., Demers L.M., Pelkman C.L., Legro R.S. (2004). A Randomized Trial of the Effects of Two Types of Short-Term Hypocaloric Diets on Weight Loss in Women with Polycystic Ovary Syndrome. Fertil. Steril..

[83] Kopp H.P., Kopp C.W., Festa A., Krzyzanowska K., Kriwanek S., Minar E., Roka R., Schernthaner G. (2003). Impact of Weight Loss on Inflammatory Proteins and Their Association With the Insulin Resistance Syndrome in Morbidly Obese Patients. Arterioscler. Thromb. Vasc. Biol..

[84] Dimitriadis G., Mitrou P., Lambadiari V., Maratou E., Raptis S.A. (2011). Insulin Effects in Muscle and Adipose Tissue. Diabetes Res. Clin. Pract..

[85] Mizgier M., Jarząbek-Bielecka G., Opydo-Szymaczek J., Wendland N., Więckowska B., Kędzia W. (2020). Risk Factors of Overweight and Obesity Related to Diet and Disordered Eating Attitudes in Adolescent Girls with Clinical Features of Polycystic Ovary Syndrome. J. Clin. Med..

[86] Melekoglu E., Goksuluk D., Akal Yildiz E. (2020). Association between Dietary Glycaemic Index and Glycaemic Load and Adiposity Indices in Polycystic Ovary Syndrome. J. Am. Coll. Nutr..

[87] Szczuko M., Malarczyk I., Zapałowska-Chwyć M. (2017). Improvement in Anthropometric Parameters after Rational Dietary Intervention in Women with Polycystic Ovary Syndrom as the Best Method to Support Treatment. Rocz. Panstw. Zakl. Hig..

[88] Herriot A.M., Whitcroft S., Jeanes Y. (2008). An Retrospective Audit of Patients with Polycystic Ovary Syndrome: The Effects of a Reduced Glycaemic Load Diet. J. Hum. Nutr. Diet..

[89] Turner-McGrievy G.M., Davidson C.R., Wingard E.E., Billings D.L. (2014). Low Glycemic Index Vegan or Low-Calorie Weight Loss Diets for Women with Polycystic Ovary Syndrome: A Randomized Controlled Feasibility Study. Nutr. Res..

[90] Seli E., Babayev E., Collins S.C., Nemeth G., Horvath T.L. (2014). Minireview: Metabolism of Female Reproduction: Regulatory Mechanisms and Clinical Implications. Mol. Endocrinol..

[91] Chavarro J.E., Rich-Edwards J.W., Rosner B.A., Willett W.C. (2009). A Prospective Study of Dietary Carbohydrate Quantity and Quality in Relation to Risk of Ovulatory Infertility. Eur. J. Clin. Nutr..

[92] Giallauria F., Palomba S., Vigorito C., Tafuri M.G., Colao A., Lombardi G., Orio F. (2009). Androgens in Polycystic Ovary Syndrome: The Role of Exercise and Diet. Semin. Reprod. Med..

[93] Sordia-Hernández L.H., Ancer Rodríguez P., Saldivar Rodriguez D., Trejo Guzman S., Servín Zenteno E.S., Guerrero González G., Ibarra Patiño R. (2016). Effect of a Low Glycemic Diet in Patients with Polycystic Ovary Syndrome and Anovulation—A Randomized Controlled Trial. Clin. Exp. Obstet. Gynecol..

[94] Dehghan M., Mente A., Zhang X., Swaminathan S., Li W., Mohan V., Iqbal R., Kumar R., Wentzel-Viljoen E., Rosengren A. (2017). Associations of Fats and Carbohydrate Intake with Cardiovascular Disease and Mortality in 18 Countries from Five Continents (PURE): A Prospective Cohort Study. Lancet.

[95] Miller V., Mente A., Dehghan M., Rangarajan S., Zhang X., Swaminathan S., Dagenais G., Gupta R., Mohan V., Lear S. (2017). Fruit, Vegetable, and Legume Intake, and Cardiovascular Disease and Deaths in 18 Countries (PURE): A Prospective Cohort Study. Lancet.

[96] Pirwany I.R., Fleming R., Greer I.A., Packard C.J., Sattar N. (2001). Lipids and Lipoprotein Subfractions in Women with PCOS: Relationship to Metabolic and Endocrine Parameters. Clin. Endocrinol..

[97] Lichtenstein A.H. (2006). Thematic Review Series: Patient-Oriented Research. Dietary Fat, Carbohydrate, and Protein: Effects on Plasma Lipoprotein Patterns. J. Lipid Res..

[98] Krauss R.M., Blanche P.J., Rawlings R.S., Fernstrom H.S., Williams P.T. (2006). Separate Effects of Reduced Carbohydrate Intake and Weight Loss on Atherogenic Dyslipidemia. Am. J. Clin. Nutr..

[99] Mizgier M., Jarząbek-Bielecka G., Wendland N., Jodłowska-Siewert E., Nowicki M., Brożek A., Kędzia W., Formanowicz D., Opydo-Szymaczek J. (2021). Relation between Inflammation, Oxidative Stress, and Macronutrient Intakes in Normal and Excessive Body Weight Adolescent Girls with Clinical Features of Polycystic Ovary Syndrome. Nutrients.

[100] Kasim-Karakas S.E., Almario R.U., Gregory L., Wong R., Todd H., Lasley B.L. (2004). Metabolic and Endocrine Effects of a Polyunsaturated Fatty Acid-Rich Diet in Polycystic Ovary Syndrome. J. Clin. Endocrinol. Metab..

[101] Dokras A., Sarwer D.B., Allison K.C., Milman L., Kris-Etherton P.M., Kunselman A.R., Stetter C.M., Williams N.I., Gnatuk C.L., Estes S.J. (2016). Weight Loss and Lowering Androgens Predict Improvements in Health-Related Quality of Life in Women With PCOS. J. Clin. Endocrinol. Metab..

[102] Stefanaki K., Karagiannakis D.S., Raftopoulou M., Psaltopoulou T., Paschou S.A., Ilias I. (2023). Obesity and Hyperandrogenism Are Implicated with Anxiety, Depression and Food Cravings in Women with Polycystic Ovary Syndrome. Endocrine.

[103] Kaur I., Suri V., Sachdeva N., Rana S.V., Medhi B., Sahni N., Ahire J., Singh A. (2022). Efficacy of Multi-Strain Probiotic along with Dietary and Lifestyle Modifications on Polycystic Ovary Syndrome: A Randomised, Double-Blind Placebo-Controlled Study. Eur. J. Nutr..

[104] Hoover S.E., Gower B.A., Cedillo Y.E., Chandler-Laney P.C., Deemer S.E., Goss A.M. (2021). Changes in Ghrelin and Glucagon Following a Low Glycemic Load Diet in Women with PCOS. J. Clin. Endocrinol. Metab..

